# Imaging features for the identification of atrial fibrillation in cryptogenic stroke patients

**DOI:** 10.1007/s00415-024-12397-y

**Published:** 2024-06-21

**Authors:** Anna Tancin Lambert, Dag Ottar Sætre, Barbara Ratajczak-Tretel, Jostein Gleditsch, Gudrun Høie, Riadh Al-Ani, Maiju Pesonen, Dan Atar, Anne Hege Aamodt

**Affiliations:** 1 Department of Neurology, Østfold Hospital Trust, Grålum, Norway; 2 Institute of Clinical Medicine, University of Oslo, Oslo, Norway; 3 Dapartment of Radiology, Østfold Hospital Trust, Grålum, Norway; 4 Department of Cardiology, Østfold Hospital Trust, Grålum, Norway; 5 Center for Biostatistics and Epidemiology, Oslo University Hospital, Oslo, Norway; 6 Department of Cardiology, Oslo University Hospital, Ullevål, Oslo, Norway; 7 Department of Neurology, Oslo University Hospital, Rikshospitalet, Sognsvannsveien 20, 0372, Oslo, Norway

**Keywords:** Atrial fibrillation, Cryptogenic stroke, Scoring system, Brain imaging

## Abstract

**Background:**

Whether specific imaging aspects can be used to identify cryptogenic stroke (CS) patients with high risk of underlying atrial fibrillation (AF) remains unclear. The purpose of this study was to evaluate brain-imaging features in CS patients and their utility as AF predictors.

**Methods:**

The Nordic Atrial Fibrillation and Stroke study was a prospective observational study of CS and transient ischemic attack patients undergoing 12-month cardiac-rhythm monitoring, biomarker and clinical assessments. In this imaging sub-study, brain magnetic resonance imaging and computed tomography scans from 106 patients were assessed for acute and chronic ischemic lesions in relation to AF occurrence and included in a score to predict AF. Receiver operating characteristics (ROC) curve was used to evaluate the discriminative ability of the score and for its dichotomization for predictive model.

**Results:**

Age, periventricular white-matter hyperintensities (PVWMH), acute lesion size, and vessel occlusion were significantly associated with AF. Acute and chronic cortical infarcts as well as chronic cerebellar infarcts were numerically more frequent in the AF group than the non-AF group. A score consisting of six features (0–6 points) was proposed (age ≥ 65 years, chronic cortical or cerebellar lesions, acute cortical lesions, PVWMH ≥ 2 in Fazekas scale, vessel occlusion, and acute lesion size ≥ 10 mm). Area under ROC curve was 0.735 and a score of ≥ 3 points was a predictor of AF.

**Conclusions:**

The suggested score was shown to identify CS patients with an increased risk of underlying AF.

## Introduction

Brain imaging findings are key elements in stroke diagnostics assessing the stroke etiology [1, 2]. Recognition of specific patterns and characteristics of ischemic lesions suggesting an embolic origin from atrial fibrillation (AF) would be beneficial for patients with cryptogenic stroke (CS). Large proportion of patients have underlying paroxysmal AF [3] and such recognition would have therapeutical implications. However, AF is difficult to uncover by standard follow-up after stroke. While the use of insertable cardiac monitors (ICMs) has been proven to be the most effective method for AF detection [4], their use is still not common in CS diagnostics. Establishing specific imaging criteria could therefore be helpful to select patients for prolonged cardiac-rhythm monitoring with ICMs. Several imaging characteristics have been demonstrated to be associated with AF such as acute cortical lesions [5], larger lesions [6], and lesions affecting multiple arterial territories [7]. Notwithstanding, studies with prolonged cardiac-rhythm monitoring showed mixed results and they focused mainly on acute lesions, while chronic lesions and white-matter hyperintensities (WMH) assessment was much less frequent.

The purpose of this study was to evaluate whether acute and chronic imaging features were associated with AF detected by ICMs during 12 months of continuous cardiac-rhythm monitoring in patients with CS, and whether a scoring system based on imaging and clinical findings could be used as a predictor of underlying AF.

## Methods

**Fig. 1:**
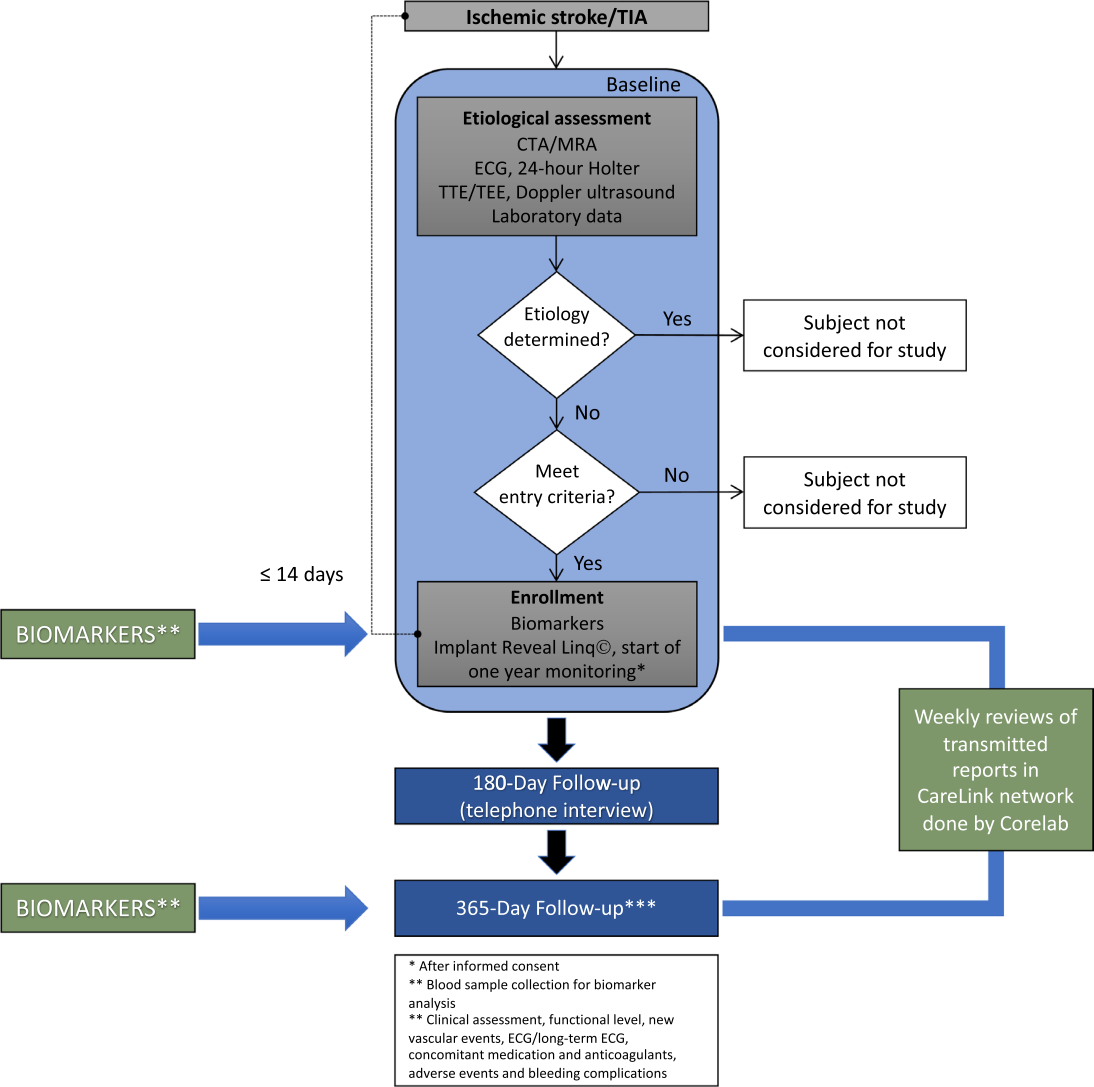
Patient flow-chart of the Nordic Atrial Fibrillation and Stroke study. *CTA* computed tomography angiography, *ECG* electrocardiography, *MRA* magnetic resonance angiography, *TEE* transoesophageal echocardiography, *TTE* transthoracic echocardiography [Modified from Ratajczak-Tretel et al. 2019 [8]]

The Nordic Atrial Fibrillation and Stroke (NOR-FIB) study was a prospective observational study of AF incidence in CS and cryptogenic transient ischemic attack (TIA) patients observed with ICMs for 12 months, performed from January 2017 to September 2021. In this single-center retrospective analysis, analyzed data were collected from patients included at one of the participating centers, Østfold Hospital Trust. Eligible patients with confirmed acute ischemia underwent a diagnostic work-up as depicted in Fig. 1 to conclude with CS or cryptogenic TIA. TIA and stroke diagnoses were used to describe the clinical presentations of the acute neurological symptoms defined as the resolution of symptoms within 24 h for TIA and the persistence of symptoms beyond 24 h for stroke. This time based definition was used to see whether patients with AF had higher tendency towards TIA and rapid resolution of symptoms than clinical stroke patients. This could address whether there are differences in the stability of embolus between AF patients and the rest of the cohort. In short, all patients including TIA patients had an acute lesion on cerebral imaging, and underwent diagnostic investigations containing general blood samples, 12-lead ECG, minimum of 24-h Holter monitoring, transthoracic echocardiography, transoesophageal echocardiography in patients ≤ 65 years of age to exclude shunting mainly from patent foramen ovale (PFO), ultrasound and computed tomography or magnetic resonance angiography to evaluate extra- and intracranial arteries, and thrombophilia screening in patients < 50 years of age. The study design and rationale have been published earlier [8] as well as the data comprising main results and ECG evaluation [9], blood biomarkers [10], and other possible underlying causes of CS beside AF after completion of one year of cardiac-rhythm monitoring [11]. CS or cryptogenic TIA were defined as an acute ischemic stroke without a determined etiology despite a thorough protocolled evaluation excluding patients with a definite etiology and source of embolism such as PFO or valvular diseases and those without adherence to participation in the study [8].

### Cardiac-rhythm monitoring

Patients included in the study had a Medtronic Reveal LINQ™ inserted within 14 days after index stroke. They obtained a CareLink monitor that enabled transmission of stored data to the CareLink Network. Two cardiologists and two neurologists (Corelab) were responsible for a weekly screening of transmitted data to CareLink for potential AF or atrial flutter (Fig. 1). Weekly evaluation secured an early AF detection and triggered a change of secondary prevention from antiplatelet agents to anticoagulants. A minimum of 2-min episode was required to establish an AF diagnosis based on the device-detection algorithm.

### Imaging analysis

As a part of the diagnostic protocol of the study, all included patients were subjected to cerebral imaging with computed tomography (CT) and/or 1.5 or 3 Tesla magnetic resonance imaging (MRI) as well as computed tomography angiography (CTA) of extra- and intracranial arteries within 14 days from symptom onset (Fig. 1). CT perfusion was done in a subset of patients. Brain CT was done routinely as the initial-imaging strategy whereas MRI was done as follow-up imaging. Brain CT-images were reconstructed to 3 mm slice thickness in the transversal plane. CTA images were reconstructed to 0.9 mm axial slices (coronal and sagittal images were made available with multiplane reformatting). For MRI the slice thickness was 4 or 5 mm according to the type of the machine and software version that was used. The following MRI sequences were obtained: Axial T2, sagittal T1, sagittal 3D FLAIR (three planes made available with multiplane reformatting), and axial diffusion. All examinations were evaluated by the same senior neuroradiologist (D.O.S.) who was blinded to clinical data and other radiological examinations. The IntelliSpace Portal v. 10 (Phillips, Eindhoven, the Netherlands) was used for the storage and the evaluation of images. MRI was chosen as the primary imaging modality for the analysis. If MRI was missing, CT and / or control CT were assessed. Acute, subacute, and chronic lesions together with the extent of white-matter lesions were recorded. Acute and subacute lesions on CT were defined as areas of hypoattenuation with associated parenchymal swelling [12]. On MRI, acute lesions were defined as having high signal intensity on diffusion-weighted imaging (DWI) and low signal intensity in apparent diffusion coefficient (ADC) maps. Subacute lesions on MRI were defined as high density signal on DWI without corresponding signal on ADC maps [13]. Due to low occurrence of subacute lesions on MRI, acute and subacute lesions were evaluated together as acute lesions in further assessments. Chronic lesions on CT were defined as areas with low density with negative mass effect [12] and on MRI as areas of low signal intensity in the presence of gliosis and cystic encephalomalacia on FLAIR images [13, 14]. To distinguish silent brain infarctions from dilated Virchow-Robin spaces and leukoaraiosis, additional criteria were used such as a lesion size of minimum 3 mm [15], specific shape, and localization [16]. All lesions were attributed to either right or left anterior circulation (internal carotid artery territory) or posterior circulation (vertebrobasilar territory) [17]. Localization of lesions was categorized as cortical, subcortical, or localized in basal ganglia, thalamus, cerebellum, and brainstem, but one lesion could affect several structures. The size of the largest acute lesion in each patient was measured as the maximum lesion diameter in axial plane on CT or on B1000 on MRI. The infarction volume, measured on B1000 sequence, was calculated as the sum of manually drawn areas in all slices affected by infarction multiplied by the slice thickness and spacing [volume = SUM of all affected areas x (slice thickness + spacing)] [18]. Acute lesions were defined as scattered if two or more lesions were localized in the same territory and as multi-territorial if at least two different arterial territories had acute lesions. The presence of fetal type of posterior communicating artery (PCOM) was noted to secure a true multi-territorial localization [19]. The amount of white-matter T2 hyperintense lesions was evaluated according to Fazekas scale dividing the white matter in periventricular and deep-white matter [20]. Each region was given a grade depending on the size and confluence of lesions: periventricular white-matter hyperintensities (PVWMH) (0 absent, 1 “caps” or pencil-thin lining, 2 smooth “halo”, 3 irregular PVWMH extending to deep-white matter) and deep-whitewhite-matter hyperintensities (DWMH) (0 absent, 1 puncate foci, 2 beginning confluence of loci, 3 large confluent areas) [20]. The scale was further dichotomized into two categories: 0 to 1 and 2 to 3 [21] as the latter category represents moderate to severe leukoaraiosis. An intracranial large-vessel occlusion (LVO) was defined as the involvement of the internal carotid artery terminus (ICA-T), the middle cerebral artery (MCA) mainstem with or without involvement of a bifurcation branch (M1), the basilar artery (BA), or the vertebral artery (VA). A medium vessel occlusion (MeVO) was defined as an isolated proximal occlusion of the MCA bifurcation branch (M2), isolated occlusions of the M3 branch, isolated occlusion of the posterior cerebral artery (PCA), and isolated occlusion of the anterior cerebral artery (ACA) A1 or A2 segments, as well as the posterior inferior cerebellar artery (PICA), the anterior inferior cerebellar artery (AICA), and the superior cerebellar artery (SCA) [22].

### Statistical evaluation

Statistical Package for Social Science (IBM SPSS Inc., version 26 for Windows) was used for statistical analysis. A *p* value < 0.05 was considered significant. Censoring of the data was performed at the time of death, dropout, or fulfilment of the study. Two patients died and did not complete the 12 months of electrocardiographic (ECG) follow-up and their AF status was assigned based on the status at dropout from the study. Differences in patient characteristics were tested using Chi-square test or Fisher’s exact test for categorical variables, and *t* test or Mann–Whitney *U* test for continuous variables. Prevalences of imaging aspects (frequency and percentages) were calculated for each group and logistic regression was used to estimate the probability of AF given each imaging aspect. Logistic regression results were presented as odds ratios and *p* values. A score consisting of the most common AF-related features in terms of patient characteristics and imaging findings and the combination of the ones with the highest discriminative ability was proposed and calculated for the patients with complete data on each feature. Receiver operating characteristic (ROC) curve was constructed for the proposed score and an optimal cut-off value dividing the population was defined in terms of optimal sensitivity and specificity. Sensitivity, specificity, positive predictive value, and negative predictive value were computed for the chosen cut-off value used for the dichotomization of the score. The dichotomized score was used as a predictor in a logistic regression model to evaluate its effect on the probability of AF. Furthermore, Kaplan–Meier curve and log-rank test were used to assess the association between the time to AF detection and proposed score.

## Results

**Fig. 2:**
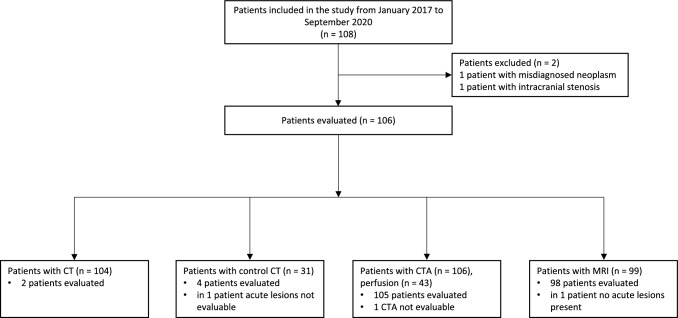
A diagram of included patients and imaging modalities used for the assessment

One hundred and six patients were included in this imaging sub-study (Fig. 2). ICMs were inserted at a median of 8 (7–11) days from the index stroke. AF or atrial flutter were detected in 31 patients (29.2%) during 12 months of continuous ECG follow-up, at a median of 12 (6–93) days. Patient characteristics are shown in Table 1. Patients with AF were significantly older, i.e., age ≥ 65 years was significantly associated with AF (*p* < 0.001). Furthermore, the AF group had significantly lower tobacco consumption and a higher pre-stroke CHA_2_DS_2_-VASC score than the non-AF group. The most prevalent stroke symptoms were numbness, weakness, and dysphasia. The latter was significantly more often seen in the AF than the non-AF group (*p* = 0.026).

**Table 1:** Patient characteristics

	All patients *n* = 106	Non-AF group *n* = 75	AF group *n* = 31	*p* value
Age (years), mean (± SD)	66.7 (± 11.4)	64.2 (± 11.5)	72.7 (± 8.8)	< 0.001*
Sex (female), *n* (%)	40 (37.7%)	26 (34.7%)	14 (45.2%)	0.311
Risk factors				
Hypertension, *n* (%)	64 (60.4%)	44 (58.7%)	20 (64.5%)	0.575
Dyslipidemia†, *n* (%)	34 (32.1%)	21 (28.0%)	13 (41.9%)	0.162
Tobacco consumption‡, *n* (%)	37 (34.9%)	31 (41.3%)	6 (19.4%)	0.031*
Heart failure§, *n* (%)	7 (6.6%)	4 (5.3%)	3 (9.7%)	0.413
Diabetes, *n* (%)	7 (6.6%)	5 (6.7%)	2 (6.5%)	1.0
Coronary disease, *n* (%)	12 (11.3%)	9 (12.0%)	3 (9.7%)	1.0
Previous stroke or TIA, n (%)	24 (22.6%)	15 (20.0%)	9 (29%)	0.312
Pre-stroke CHA_2_DS_2_-VASc, median (IQR)	2 (1–4)	2 (1–4)	2 (2–4.5)	0.047*
Acute treatment				
IV tPA treatment, *n* (%)	29 (27.4%)	19 (25.3%)	10 (32.3%)	0.467
Thrombectomy, *n* (%)	5 (4.7%)	4 (5.3%)	1 (3.2%)	1.0
Time to IV tPA (minutes), median (IQR)	118 (86.5–133.5)	120 (104–140)	88 (60–117)	0.053
Stroke severity				
NIHSS admission, median (IQR)	1.5 (0–3)	1 (0–3)	2 (1–3.5)	0.202
NIHSS discharge, median (IQR)	1 (0–2)	1 (0–2)	1 (0–1.5)	0.827
mRS admission, median (IQR)	0 (0–0)	0 (0–0)	0 (0–0)	0.665
mRS discharge, median (IQR)	1 (0–2)	1 (0–1)	1 (0.5–2)	0.581
Clinical presentation				
TIA *n* (%)	25 (23.6)	17 (22.7%)	8 (25.8%)	0.729
Stroke *n* (%)	81 (76.4%)	58 (77.3%)	23 (74.2%)	
Stroke symptoms admission				
Numbness or weakness *n* (%)	80 (75.5%)	57 (76.0%)	23 (74.2%)	0.844
Dysphasia *n* (%)	54 (50.9%)	33 (44.0%)	21 (67.7%)	0.026*
Visual impairment *n* (%)	9 (8.5%)	9 (12.0%)	0 (0.0%)	0.056
Trouble walking, dizziness, loss of balance or coordination *n* (%)	33 (31.1%)	22 (29.3%)	11 (35.5%)	0.534
Severe headache without known cause *n* (%)	9 (8.5%)	7 (9.3%)	2 (6.5%)	1.0
Confusion *n* (%)	6 (5.7%)	2 (2.7%)	4 (12.9%)	0.059

*AF* Atrial fibrillation, *CHA2DS2-VASc* Congestive heart failure, Hypertension, Age ≥ 75 years (doubled), Diabetes mellitus, prior Stroke or TIA or thromboembolism (doubled), Vascular disease, Age 65 to 74 years, Sex category, *IQR* Interquartile range, *IV tPA* Intravenous tissue plasminogen activator mRS, modified Rankin Scale, *n* Number, *NIHSS* National Institutes of Health Stroke Scale, *SD* Standard deviation, *TIA* Transient ischemic attack

*Significant results

†Self-reported or use of medication on admission

‡Current smoking/ snuff usage or if cessation within 1 year before stroke

§Self-reported, stated in patient journal or ejection fraction ≤ 40% on echocardiography performed as a part of diagnostics

One hundred and four patients (98.1%) underwent brain CT and 99 patients (93.4%) brain MRI. CT perfusion was done in 43 patients (40.6%). The number of patients included in the analysis and the imaging techniques used are shown in Fig. 2. Evaluated imaging (Fig. 2) was performed for CTA at a median of 1 (0 − 5) days, for MRI at a median of 2 (1 − 3) days, and for CT or control CT within 1 day from the onset of symptoms. Almost half of the included patients had verified chronic ischemia on brain imaging (Table 2), although only 22.6% had known history of previous stroke or TIA (Table 1) (only one patient with solely TIA). Within the group of patients with chronic lesions, patients with AF had more often prior clinical stroke or TIA than those without AF (57.1% vs 27.8%, *p* = 0.052). Chronic lesions were almost equally distributed in the studied population between anterior and posterior circulation, while acute lesions were predominantly located in the anterior circulation. Approximately 1 out of 3 patients had Fazekas scale > 1.

**Table 2:** Evaluated imaging features

All patients *N* = 106				
Evaluated imaging modality				
MRI	99 (93.4%)			
CT	7 (6.6%)			
Chronic lesions	50 (47.2%)	Acute lesions		104 (98.1%)
Number of lesions ≥ 2 lesions	31 (29.2%)	Number of lesions 1 − 9 10 − 19 ≥ 20		78 (73.6%) 22 (20.8%) 4 (3.8%)
Territory		Territory		
Anterior Right Left Bilateral	30 (28.3%) 13 (12.3%) 7 (6.6%) 10 (9.4%)	Anterior Right Left Bilateral		81 (76.4%) 33 (31.1%) 35 (33.0%) 13 (12.3%)
Posterior	35 (33.0%)	Posterior		38 (35.8%)
		Multi-territorial		23 (21.7%)
Localization of chronic infarctions		Localization of acute infarctions		
Cortical	16 (15.1%)	Cortical		69 (65.1%)
Subcortical	26 (24.5%)	Subcortical		72 (67.9%)
Basal ganglia	4 (3.8%)	Basal ganglia		22 (20.8%)
Thalamus	6 (5.7%)	Thalamus		6 (5.7%)
Cerebellum	29 (27.4%)	Cerebellum		19 (17.9%)
Brainstem	3 (2.8%)	Brainstem		5 (4.7%)
		Scattered		50 (47.2%)
WMH score		Hemorrhagic transformation		2 (1.9%)
PVWMH 0 − 1 2 − 3	67 (63.2%) 39 (36.8%)			
DWMH 0 − 1 2 − 3	71 (67.0%) 35 (33.0%)			

*CT* Computed tomography, *DWMH* Deep-whitewhite-matter hyperintensities, *MRI* Magnetic resonance imaging, *PVWMH* Periventricular white-matter hyperintensities, *WMH* White-matter hyperintensities

Patients with AF had more acute ischemic lesions with diameter ≥ 10 mm (OR 3.52 95% CI 1.11 − 11.18), and more often MeVO (OR 3.73 95% CI 1.23 − 11.32) than patients without AF. After Fazekas scale categorization, PVWMH score ≥ 2 was associated with almost three times higher odds of AF than lower score (OR 2.93 95% CI 1.23 − 6.95) (Table 3). Multi-territorial ischemic lesions were slightly more frequent in patients without AF (Table 3). However, there were no significant differences in acute and chronic lesions between the groups. (Table 3). Additional three patients had a vessel occlusion detected, though these were evaluated as chronic occlusions and were therefore not included in calculations.

**Table 3:** Between group differences for evaluated imaging features

	Non-AF group	AF group	OR (95% CI)	*p* value
Chronic lesions	*n* = 75	*n* = 31		
Lesions present	36 (48.0%)	14 (45.2%)	0.89 (0.39 − 2.07)	0.790
Vascular territory, *n* (%)				
Anterior circulation	23 (30.7%)	7 (22.6%)	0.66 (0.25 − 1.75)	0.402
Right	11 (14.7%)	2 (6.5%)	0.40 (0.08 − 1.93)	0.254
Left	5 (6.7%)	2 (6.5%)	0.97 (0.18 − 5.26)	0.968
Bilateral	7 (9.3%)	3 (9.7%)	1.04 (0.25 − 4.32)	0.956
Posterior circulation	22 (29.3%)	13 (41.9%)	1.74 (0.73 − 4.15)	0.212
Localization of chronic infarctions, *n* (%)				
Cortical	11 (14.7%)	5 (16.1%)	1.12 (0.35 − 3.54)	0.848
Subcortical	19 (25.3%)	7 (22.6%)	0.86 (0.32 − 2.31)	0.765
Basal ganglia	3 (4.0%)	1 (3.2%)	0.80 (0.08 − 8.00)	0.849
Thalamus	3 (4.0%)	3 (9.7%)	2.57 (0.49 − 13.51)	0.264
Cerebellum	19 (25.3%)	10 (32.3%)	1.40 (0.56 − 3.51)	0.468
Brainstem	2 (2.7%)	1 (3.2%)	1.22 (0.11 − 13.93)	0.875
WMH score				
PVWMH 0–1 2–3	53 (70.7%) 22 (29.3%)	14 (45.2%) 17 (54.8%)	Reference 2.93 (1.23 − 6.95)	0.015*
DWMH 0–1 2–3	53 (70.7%) 22 (29.3%)	18 (58.1%) 13 (41.9%)	Reference 1.74 (0.73 − 4.15)	0.212
Acute lesions	*n* = 74	*n* = 30		
Vascular territory, *n* (%)				
Anterior circulation	56 (75.7%)	25 (83.3%)	1.61 (0.54 − 4.82)	0.397
Right	21 (28.4%)	12 (40.0%)	1.68 (0.69 − 4.09)	0.251
Left	25 (33.8%)	10 (33.3%)	0.98 (0.40 − 2.41)	0.965
Bilateral	10 (13.5%)	3 (10.0%)	0.71 (0.18–2.79)	0.625
Posterior circulation	30 (40.5%)	8 (26.7%)	0.53 (0.21 − 1.36)	0.187
Multi-territorial	17 (23.0%)	6 (20.0%)	0.84 (0.30 − 2.39)	0.741
Localization of acute infarctions, *n* (%)				
Cortical	46 (62.2%)	23 (76.7%)	2.00 (0.76 − 5.26)	0.160
Subcortical	50 (67.6%)	22 (73.3%)	1.32 (0.51 − 3.39)	0.564
Basal ganglia	16 (21.6%)	6 (20.0%)	0.91 (0.32 − 2.60)	0.854
Thalamus	5 (6.8%)	1 (3.3%)	0.48 (0.05 − 4.25)	0.506
Cerebellum	15 (20.3%)	4 (13.3%)	0.61 (0.18 − 2.00)	0.410
Brainstem	4 (5.4%)	1 (3.3%)	0.60 (0.07 − 5.63)	0.658
Scattered	36 (48.6%)	14 (46.7%)	0.92 (0.40 − 2.16)	0.855
Lesion number	*n* = 74	*n* = 30		
< 10 10 − 19 ≥ 20	56 (75.7%) 15 (20.3%) 3 (4.1%)	22 (73.3%) 7 (23.3%) 1 (3.3%)	Reference 1.12 (0.43 − 3.31) 0.85 (0.08 − 8.60)	0.742 0.889
Lesion diameter				
< 10 mm n (%) ≥ 10 mm n (%)	26 (35.1%) 48 (64.9%)	4 (13.3%) 26 (86.7%)	Reference 3.52 (1.11 − 11.18)	0.033*
Lesion volume				
< 5 mL ≥ 5 mL	56 (75.7%) 18 (24.3%)	18 (60.0%) 12 (40.0%)	Reference 2.07 (0.84 − 5.12)	0.113
Vessel occlusion†	16 (21.6%)	11 (35.5%)	2.00 (0.79 − 5.01)	0.142
None	58 (78.4%)	20 (64.5%)	Reference	
MeVO	7 (9.5%)	9 (29.0%)	3.73 (1.23 − 11.32)	0.020*
LVO	9 (12.2%)	2 (6.5%)	0.64 (0.13 − 3.24)	0.594

*AF* Atrial fibrillation, *DWMH* Deep-whitewhite-matter hyperintensities, *LVO* Large-vessel occlusion, *MeVO* Medium vessel occlusion, *mL* Mililiter, *mm* Millimetres, *PVWMH* Periventricular white-matter hyperintensities, *WMH* White-matter hyperintensities

*Significant results

†Evaluated in 105 patients

**Fig. 3:**
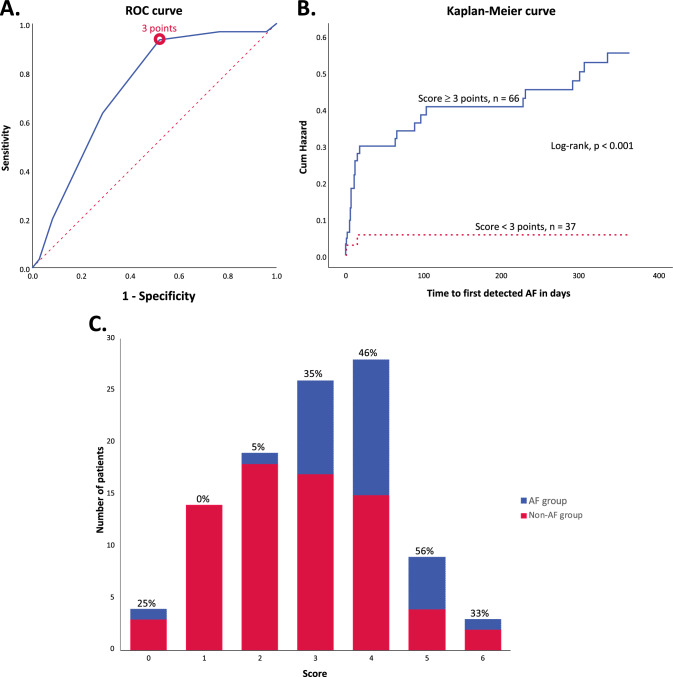
Panel A shows the Receiver operating characteristics (ROC) curve for the proposed AF prediction score and the optimal cut-off value (circle). Panel B shows the Kaplan–Meier curve for subgroups of patients defined by the dichotomized score, cumulative hazard of atrial fibrillation (AF) in these groups, and association with the time to first verified episode of AF. Panel C shows the number of patients with each score value (height of the bars), grouped by the AF status. The proportion (%) of AF-positive patients for each score is given above each bar

Based on the patient characteristics and imaging analyses, a score for AF detection was constructed consisting of characteristics that were more common in the AF group than the non-AF group, the combination of which reached the highest discriminative ability. The score consisted of six features, each contributing 1 point to the total score (range 0–6 points); chronic cortical or cerebellar lesions, acute cortical lesions, acute lesion size ≥ 10 mm, PVWMH score ≥ 2, vessel occlusion, and age ≥ 65 years. (Table 4). A score of three points was chosen as an optimal cut-off value to divide the population (Fig. 3A). The AUC for the proposed score was 0.735 (0.634 − 0.836). Including symptoms of dysphasia slightly improved its discriminative ability, (AUC 0.752 vs 0.735), as well as inclusion of only MeVO (AUC 0.753 vs 0.735). The chosen cut-off gave a sensitivity of 93.3% (95% CI 77.9 − 99.2%), a specificity of 47.9% (95% CI 36.1 − 60.0%), a PPV of 42.4% (95% CI 30.3 − 55.2%), and an NPV of 94.6% (95% CI 81.8 − 99.3%). Patients with score ≥ 3 points had 12.9 times higher the odds of AF 95% CI (2.86 − 58.15) in comparison to the patients with a lower score. In this population, the AF-detection rates reached 42.4%, while in the population with lower scores this was only 5.4% (only two patients with AF). Based on the log-rank test, score ≥ 3 points was associated with higher cumulative hazard of AF as well as its earlier detection, *p* < 0.001 (Fig. 3B). The proportions of patients for each score value are shown in Fig. 3C, grouped by AF status. Including dysphasia in the proposed score increased the AUC but changed the cut-off value to 4 points, resulting in a lower sensitivity and NPV, 83.3% and 90.0% respectively. For score including MeVO only, the sensitivity and NPV were the same as for the proposed score with all-vessel occlusions.

**Table 4:** Proposed score to predict underlying AF in CS and cryptogenic TIA patients

Feature	Points
Chronic cortical or cerebellar lesions	1
Acute cortical lesions	1
Acute lesion size ≥ 10 mm	1
PVWMH score ≥ 2	1
Vessel occlusion	1
Age ≥ 65 years	1
Total score	6

## Discussion

In our study, CS and cryptogenic TIA patients with AF detected by ICMs within 12-month follow-up had more often PVWMH, acute ischemic lesions with diameter ≥ 10 mm, and MeVO than patients without AF. Other previously described imaging findings believed to predict AF such as acute cortical and multi-territorial stroke or LVO were not significantly associated with AF in the studied population. However, the combination of specific imaging and clinical features showed a good ability to predict AF in patients with CS and cryptogenic TIA.

The effectiveness and usefulness of prolonged cardiac-rhythm monitoring for AF detection in CS patients have been shown in multiple studies and meta-analyses [3, 4, 23], but only a few of them focused on brain imaging and its utility in AF prediction (Table 5). In a recent meta-analysis, bilateral and multi-territorial lesions on MRI in patients with acute ischemic stroke were associated with cardioembolic stroke [24]. In our study, neither of these characteristics were significantly different between patients with and without AF which is in line with the results from other CS studies using prolonged cardiac-rhythm monitoring (Table 5). Acute cortical lesions represent another imaging characteristic shown to be predictive of AF in multiple studies [5, 21, 25]. In our cohort, this trend was also observed, however this was not statistically significant. These results are similar to other studies (Table 5) except for a study by Kass-Hout et al. that found association between cortical lesions and underlying AF [26]. In terms of the size of acute lesions, several cut-off values for lesion diameter have been used in studies with prolonged cardiac monitoring such as 5 mm [27] or 20 mm [28] and studies proposing scoring systems with a diameter over 4 cm [6]. The total size and volume of acute ischemic lesions in our study was lower than size and volume seen in a general AF-stroke population which most likely reflects the inclusion of patients with low disability and mild to moderate strokes in CS studies with prolonged monitoring [28–30]. In our population, we chose a diameter of 10 mm as it divided the population best and performed well as a part of the proposed score.

**Table 5:** Earlier studies describing brain-imaging features in cryptogenic stroke patients assessed with prolonged cardiac-rhythm monitoring

Study	Population		Cardiac rhythm and length of monitoring	AF rate	Imaging modality	Acute lesions	Chronic lesions	Leukoairosis/WMH
	Type	Size (*n*)						
Gaillard et al., 2010 [43]	Non-CES, TIA (83% with cryptogenic events)	98	Transtelephonic ECG (at least 1 ECG per day for 1 month)	9.2% (8.5% for CS)	MRI	Association with non-lacunar anterior circulation infarction (*p* = 0.03), in multivariable analysis OR 9.9 (95% CI 1.1–90.6), *p* = 0.042	Not stated	Not stated
Bhatt et al., 2011 [44]	CS	50	MCOT (28 days)	24%	MRI	High signal on DWI predicted AF OR 4.3 (95% CI 5–36.3), *p* = 0.041 78.6% of patients with AF had multiple DWI signals OR 11.1 (95% CI 2.5–48.5), *p* < 0.01	Not stated	Not stated
	TIA	12						
Rabinstein et al., 2013 [45]	CS	64	MCOT (21 days)	25%	MRI	Ns for pre-defined embolic pattern (wedge-shaped lesions of cortex, multiple, bilateral or multi-territorial infarctions) in patients < 65 years, AF observed just in CS group (*p* = 0.03)	Not stated	Not stated
	stroke of known cause	64		14%				
Favilla et al., 2015 [31]	CS	179	MCOT (28 days)	14% (58% with AF ≥ 30 s)	CT or MRI	Ns for multi-territorial or wedge-shaped cortical infarction	Cortical or cerebellar infarction (*p* = 0.021), in multivariable analysis adjusted for age > 60 years, sex and race OR 5.6 (95% CI 1.4–22), *p* = 0.013 for AF ≥ 30 s	Not stated
	TIA	48						
Bernstein et al., 2015 CRYSTAL AF [27]	CS, TIA	212	ICM (12 months)	12.4%	CT or MRI	Ns for specific localization of infarctions	Any chronic lesion HR 2.84 (95% CI 1.13–7.15), *p* = 0.02 Borderline association with chronic territorial lesions HR 2.37 (95% CI 0.98–5.72), *p* = 0.05)	*p* < 0.01.HR 2.94 (95% CI 1.28–6.71)
Sudacevschi et al., 2018 [30]	CS, TIA	171 (84% with CS)	Holter (21 days)	15%	MRI	Tendency toward multiple lesions and small lesions in non-lacunar territories (*p* = 0.05) Ns for affection of both anterior and posterior circulation or of both hemispheres	Not stated	*p* = 0.001 in multivariable analysis OR 4.2 (95% CI 1.2–15.6)
Kass-Hout et al., 2018 [26]	CS, TIA	132 (72% with CS)	MCOT (mean 25 days)	13%	MRI	1 ≥ cortical lesions OR 5.2, (95% CI 1.3–19.0), *p* = 0.01 In multivariable analysis OR 5.5 (95% CI 1.4–20.9)	Not stated	Not stated
Vollmuth et al., 2019 [28]	CS, TIA with definite cortical syndrome	104	ICM (median 674.5 days)	20.2%	MRI	ns for localization, size, scattering or multi-territorial stroke	ns for rate of chronic lesions, distribution, or number of affected territories	Ns
		166	Long-term ECG (72 h and at least one 24-h Holter)	100%				*p* < 0.05 compared to AF with ICM *p* < 0.001 compared to no AF with ICM

*AF* atrial fibrillation, *CI* confidence interval, *CS* cryptogenic stroke, *DWI* diffusion-weighted imaging, *ECG* electrocardiogram, *HR* hazard ratio, *ICM* insertable cardiac monitor, *MCOT* mobile cardiac outpatient telemetry, *MRI* magnetic resonance imaging, *n* number, *non-CES* non-cardioembolic stroke, *ns* non-significant, *OR* odds ratio, *p* *p* value, *TIA* transitory ischemic attack

Only a few CS studies described ischemic lesions [27, 28, 30]. No significant association was found between chronic lesions and AF detection in our study. Bernstein et al. found that the presence of any chronic lesion was associated with AF [27], and Favilla et al. found an association particularly for cortical or cerebellar stroke [31]. Even though not significant, patients with AF in our study had more often chronic cortical and cerebellar lesions than those without AF particularly when both localizations were affected. The importance of the evaluation of chronic lesions was also emphasized by the fact that the frequency of chronic lesions in our cohort was two times higher than the frequency of clinical stroke or TIA cases. Simultaneously, clinical stroke or TIA were more often seen in patients with AF than those without. WMH is also a feature that is not often analyzed as it has mostly been related to small vessel disease rather than AF. Presence of any degree of WMH was not associated with AF in our study, but if categorized to PWMH and DWMH [21], more advanced PWMH (score ≥ 2) was predictive of AF. Results from other studies are inconclusive in terms of the differences between PWMH and DWMH in relation with AF [32, 33], and the general contribution and role of AF in WMH development [34]. Nevertheless, several CS studies with prolonged cardiac monitoring [27, 28, 30] as well as population studies found a strong association with AF [32, 35].

Large and medium vessel occlusions have been shown to be predictive of AF in several studies [29, 36, 37]. In our population, vessel occlusion was numerically more frequent in AF than non-AF group. Particularly, MeVO was significantly associated with AF, and patients with MeVo had almost four times increased odds of AF. However, there were also some patients with probable chronic occlusions that were not included in the calculations. In the proposed score both proximal and distal vessel occlusions were included to make the score more applicable for clinical use. Most studies with prolonged monitoring did not focus on vessel occlusions. Sudacevschi et al. did not find association between occlusions or stenosis detected on MRI and AF [30]. However, Doijiri et al. found both LVO and M2 and M3 occlusions to be an independent predictor of AF [38].

Generally, our study showed similar results to other comparable studies. The main differences with other studies were shorter cut-offs for AF duration at 30 s [28, 31, 39] or even shorter [31], later initiation and shorter length of monitoring (Table 5), and also inclusion of patients with PFO [28, 39] that had been shown to have another lesion pattern than AF-related stroke [40].

Based on the results from our and previous studies, individual imaging findings alone are likely not robust enough for AF prediction. Development of reliable scores consisting of several imaging criteria as well as clinical characteristics could be helpful in recognizing patients with underlying AF and secure wider availability of ICMs for CS patients. Our suggested score was comparable to other clinical scores in terms of predictive ability for AF with AUC of 0.735 [41] with sensitivity and NPV over 90% for three or more features resulting in 12 times higher probability of AF than in those with less but it needs further validation in another CS cohort. Proposed aspects are also feasible in terms of evaluation by physicians treating CS patients. At 12-month follow-up, other possible etiologies were discovered such as malignancy or antiphospholipid syndrome that could also present with characteristics similar to AF such as multi-territorial stroke [42]. However, this represents a normal-CS population that also consists of other diagnoses beside AF which can be revealed after some time [11]. Consequently, no patients were excluded from the analysis, even if a probable etiology was revealed after 1 year of follow-up only.

### Strengths and limitations

All patients included in the study underwent very thorough diagnostic work-up before the diagnosis of CS was made. Furthermore, all patients were followed extensively for underlying AF for a period of 12 months and continuous cardiac-rhythm monitoring was initiated early after the index stroke. A large number of radiological parameters, including specifications on the extension of WMH and the size of acute lesions, were evaluated by an experienced neuroradiologist blinded to clinical data. Furthermore, lesions were attributed to all affecting structures, not just one. In addition, fetal PCOM was noted in all patients to correctly identify multi-territorial stroke.

One of the limitations is that only the largest ischemic lesions were measured due to the large number of acute lesions that were mainly very small in size and present in only few CT or MRI slices. However, all the consequent lesions were smaller in size and categorization of patients based on a specific diameter and volume cut-off unified the data and was performed also in other studies [27, 28]. Some studies found that even patients with AF can have small subcortical lesions, although lacunar stroke was an exclusion criterion in our cohort since the etiology in these patients was determined. A large proportion of AF patients in our study had lesions affecting subcortex, however particular measurements of solely subcortical lesions were not performed. Another limitation is that only one neuroradiologist performed the blinded assessments without an additional evaluator.

## Conclusion

Combining imaging and clinical characteristics in a prediction score consisting of 6 features (age ≥ 65 years, chronic cortical or cerebellar lesions, acute cortical lesions, PVWMH ≥ 2, vessel occlusion, and acute lesion size ≥ 10 mm) performed well to discriminate between AF- and non-AF patients. The presence of three or more features predicted underlying AF in this cryptogenic stroke and TIA population.

## Data Availability

The data that support the findings of this study are available from the corresponding author upon reasonable request.
